# The nature of UK supermarkets’ policies on checkout food and associations with healthfulness and type of food displayed: cross-sectional study

**DOI:** 10.1186/s12966-018-0684-2

**Published:** 2018-06-11

**Authors:** Katrine T. Ejlerskov, Martine Stead, Ashley Adamson, Martin White, Jean Adams

**Affiliations:** 1 Centre for Diet and Activity Research, MRC Epidemiology Unit, University of Cambridge, Cambridge, UK; 2 Institute for Social Marketing, Faculty of Health Sciences and Sport, University of Stirling, Stirling, UK; 3 Institute of Health & Society and Human Nutrition Research Centre, Newcastle University, Newcastle upon Tyne, UK

**Keywords:** Checkout, Supermarket, Nutrition, Obesogenic environment, Marketing, Snack food, Policy, Unhealthy, Public health

## Abstract

**Background:**

Food choices are often determined by stimuli from our immediate surroundings, including strategic placement in shops to encourage impulse purchases. One example of this is food in shop checkout areas. Recently a number of UK supermarkets have voluntarily committed to providing healthier checkout foods. The aim of this study was to document the nature of current UK supermarket checkout food policies; determine whether there are any differences in the healthfulness and type of food displayed at checkouts in supermarkets according to the presence or nature of policies; and determine whether supermarkets are adhering to their checkout food policies.

**Methods:**

Survey of checkout food policies. Cross-sectional observations in 69 supermarkets (covering 14 store formats) in the East of England in Feb-May 2017 of the number and type of checkout foods on each ‘checkout journey’ (each possible route through the checkout area). Checkout foods were categorised as less healthy or healthier, using the UK Food Standard’s Agency’s Nutrient Profile Model, and into food groups. Checkout food policies were categorised as clear and consistent, vague or inconsistent, or absent.

**Results:**

Checkout food policies differed between store formats in some supermarket groups. Across the 14 store formats included, two had no checkout food policy, six had ‘clear and consistent’ policies, and six ‘vague or inconsistent’ policies. In supermarkets with clear and consistent policies there were a median of 13 products per checkout journey, of which 35% were less healthy. Comparable figures for supermarkets with vague or inconsistent, and absent policies were 15 (57%) and 39 (90%) respectively (ps for trend < 0.001).

Whilst most supermarkets with a clear and consistent checkout food policy were fully adherent to their policy, those with vague or inconsistent policies were not.

**Conclusions:**

Most UK supermarkets have checkout food policies, but not all are clear and consistent. Supermarkets with clear and consistent policies display fewer checkout foods and a lower proportion of these are less healthy than in other supermarkets. Supermarkets with clear and consistent policies adhere well to these. More stores should be encouraged to develop a clear and consistent checkout food policy. This may require non-voluntary intervention.

**Electronic supplementary material:**

The online version of this article (10.1186/s12966-018-0684-2) contains supplementary material, which is available to authorized users.

## Background

Food choices are often determined by stimuli from our immediate surroundings [1, 2]. Thus, everyday exposure to energy-dense, nutrient-poor foods contributes to excess energy consumption and obesity [3–5]. One commonly encountered food stimulus is the strategic placement of food and non-alcoholic drinks (which we refer to throughout as ‘food’) to encourage unplanned, impulse, purchases [6, 7]. An example of this is food placed in store checkout areas. Food in checkout areas has been found to lead to impulse purchases and child purchasing requests [6–10], which parents find hard to resist [9, 11–13]. Around 80% of UK checkout foods were considered unhealthy in 2014–15 [14, 15].

In recent years, the display of unhealthy foods at supermarket checkouts and non-food stores has gained attention from media [16, 17], advocacy groups [11, 12, 18–20] and researchers in Australia [8–10, 21], the UK [14, 15], the US [21–25], Denmark [21, 26], Norway [27, 28], Canada, Sweden and the Netherlands [21]. Most research has been descriptive, demonstrating large quantities of checkout food which tends to be less healthy [8–10, 14, 15, 21–24]. There have also been a number of researcher-led intervention studies in both supermarkets and other environments [25–28]. These vary substantially in terms of the products that are removed from checkouts, replacement products, and reported effects on purchases [25–30]. Research in more controlled settings has found that the balance of “healthy” to “less healthy” foods displayed at checkouts influences customers’ behaviour, with healthy foods being more likely to be selected when they were in the majority [30].

Alongside researcher-led interventions, supermarket-led voluntary pledges and government-led regulation of checkout food have also been proposed [8, 11, 20]. In response to consumer concern, campaigns and negative media coverage, some UK supermarkets have voluntarily committed to providing healthier food at their checkouts. Whilst we are aware of UK supermarket checkout food policies from as early as 1994 [31], most have been made in the last 5 years. These policies are, without exception, framed in an open-ended manner with no specified end dates.

The impact of such policies has not yet been evaluated. In particular, it is not clear what the nature of supermarket checkout food policies are, whether the presence or nature of these is associated with different types of checkout food, or whether supermarkets adhere to their own policies.

The aims of our research were therefore to: (i) document the nature of current UK supermarket checkout food policies; (ii) determine whether there are any differences in the healthfulness and type of food displayed at checkouts in UK supermarkets according to the presence or nature of policies; and (iii) determine whether UK supermarkets are adhering to their own checkout food policies.

## Methods

To address our first aim, we conducted a survey of current supermarket checkout food policies in the UK. To address our second and third aims, we conducted in-store observations of checkout foods in nine leading UK supermarket groups in February–May 2017. As UK supermarket checkout food policies are voluntary and supermarket-specific, this cross-sectional design allowed us to take advantage of ‘natural’ variation in the presence and nature of policies to undertake comparative analyses. The STROBE guidelines for reporting observational studies were followed [32].

### Selection of supermarket groups for inclusion

Nine large national supermarket groups, representing more than 90% of UK grocery market share [33], were included: Aldi, Asda, Coop, Lidl, M&S, Morrisons, Sainsbury’s, Tesco, and Waitrose. The remaining UK supermarket groups are either highly specialised (frozen foods only), only online, or smaller independent franchise chains. Some of the included groups include stores across a range of formats (e.g. large out of town hypermarkets, local convenience stores). Where this was the case, we included all formats in included groups.

As our intention was to study the nature and impact of checkout food policies, rather than ‘name and shame’ particular supermarket groups, we have chosen not to identify specific supermarket groups in the results section of this paper.

### Identification of checkout food policies

To document the presence and nature of checkout food policies, we first searched supermarket groups’ annual reports, webpages and press releases for relevant information. If we failed to find relevant or detailed enough information in these sources, we contacted supermarkets’ customer services via email, phone or letter. As a last resort, we used information in newspaper articles or similar secondary sources. In some instances supermarket groups had different policies for different store formats within their group.

We found that checkout food policies varied in terms of: how clear, or specific, they were regarding which products were to be removed and what they were to be replaced with; and how consistently they applied to all checkouts. Thus, policies were categorised as: ‘clear and consistent’ (policies that provided detailed information on which products should be removed and examples of replacement products, and which applied to all checkouts within a supermarket group or format); ‘vague or inconsistent’ (less specific information on products to be removed or introduced, or did not apply to all checkouts) or “No policy” (See Table 1 for details).

**Table 1:** UK supermarket policies on checkout food, May–July 2017

Supermarket group	Store format	Products removed	Suggested replacement products	Information source	Checkouts where policies apply	Year of implementation (month if known)	Policy category
1	Hypermarket Large supermarket Small supermarket Convenience store	Sweets and chocolate	Healthier snacks such as dried fruit, nuts and cereal bars. These should either be one of your 5-a-day, have no ‘red’ traffic light ratings, be in calorie controlled snack packs, or be deemed by the Department of Health to be a ‘healthier snack’.	Annual report, supermarket’s own web page	All checkouts	2015 (January)	Clear and consistent
2	Large supermarket	Confectionery	Not stated	Customer service	Main checkouts where a family is expected to shop with a trolley	2004	Vague or inconsistent
	Convenience store	No policy	No policy	Customer service	No policy	NA	No policy
3	Large supermarket	Limits display of confectionery treats to one in three checkouts	“Guilt free” checkouts (not defined)	Radio interview and consumer report	1/3 of checkouts	2012	Vague or inconsistent
4	Large supermarket	Sweets and chocolate	Range of alternative snacks, including fruit, nuts and bottled water	Supermarket’s own web page, customer service	Main bank checkouts (directly at the till)	2016 (February)	Vague or inconsistent
5	Large supermarket	Confectionery, chocolate and sweets	Healthier options including dried fruit, nuts, juices and water	Supermarket’s own web page	All checkouts	2015 (January)	Clear and consistent
6	Convenience store	No policy	No policy	Customer service	No policy	NA	No policy
7	Large supermarket	Sweets	Not stated	Customer service	All checkouts	2014 (August)	Vague or inconsistent
	Convenience store	Sweets	Not stated	Customer service	All checkouts, except in stores with petrol stations	2014 (August)	Vague or inconsistent
8	Large supermarket	Sweets and chocolate	More nutritious options such as dried fruits and nuts, seeds, fresh fruit and fruit juices	Supermarket’s own web page	All checkouts	2014 (January)	Clear and consistent
9	Convenience store	Confectionery, crisps, cakes and biscuits	Not stated	Customer service	All checkouts in stores owned by company, not in franchise stores	2015 (September)	Vague or inconsistent

### Selection of stores for inclusion in the cross-sectional survey

To determine whether checkout food varied according to policies, and whether supermarkets adhered to their policies, we conducted in-store observations. All stores in the included supermarket groups in the East of England region were identified using Ordnance Survey’s Points of Interest database (*n* = 978, June 2015) [34]. This has previously been found to have reasonable agreement with field observations for identification of food outlets [35, 36].

We aimed to include five stores of each format within each included supermarket group, and selected stores for inclusion using stratified random sampling. In one case there were only four stores of a particular format in the East of England region therefore all four stores were included.

### Definition of checkout areas and checkout food

Checkout areas were defined as “any compulsory areas that shoppers had to pass through to pay for their goods”, as defined in previous studies [14, 15]. This included self-service checkouts, self-scan checkouts and payment points placed anywhere in stores. Food and non-alcoholic drink products within arm’s reach (approc. 70 cm) of any point from where customers entered, to where they exited the checkout area was defined as checkout food.

### Collection of data on the range of checkout foods present

In-store observations were conducted using methods we have previously developed [14]. A fieldworker was trained by an experienced researcher in one supermarket not included in the study sample, prior to the data collection. The fieldworker visited all sampled stores and recorded all checkout foods present by talking into a discreet, mobile-phone type, voice recorder. Supermarket managers were not asked for permission to make these in-store observations. Recordings were transcribed within a week of data collection. Only the type of products displayed was recorded, not packaging size or number of shelf ‘facings’. We have previously found this method to have high inter-rater reliability [14].

### Categorisation of the healthfulness of checkout food

To determine the healthfulness of supermarket checkout food, we collected nutritional information on all checkout foods observed. We used this to determine whether foods were ‘less healthy’ or not according to the UK Food Standards Agency’s Nutrient Profile Model (NPM) [37]. This model assigns positive points based on energy, sugar, fat and sodium content of products, while negative points are assigned for protein, fibre, fruit, vegetable and nut content. A food is classified as “less healthy” where it scores 4 points or more, while a drink is classified as “less healthy” if it scores 1 point or more.

Nutritional information was obtained from one of a number of sources. We gave preference to information on manufacturers’ websites, followed by supermarket websites, followed by food packaging. When no information could be found from any of these sources, information on an equivalent product in the UK Nutrient Databank was imputed. The nutritional information collected was: energy, fat, saturated fat, total carbohydrates, sugar, sodium/salt, protein, fibre, and fruit, nuts and vegetable content, all per 100 g.

The Food Standards Agency’s NPM was developed to identify foods that cannot be advertised on television to children and is thus policy-relevant in the UK context. The model balances the content of ‘positive’ versus ‘negative’ nutrients in foods and uses this to calculate an overall score. Standard cut-offs are used to categorise food and non-alcoholic drinks as ‘less healthy’ or ‘healthier’. This NPM has been found to have reasonable specificity and sensitivity [38].

### Categorisation of the type of checkout foods

To characterise the type of checkout foods observed, we used the food groups used in the UK National Diet & Nutrition Survey (NDNS) [39], again chosen because these are policy relevant in the UK context. Only those food groups from which we found checkout foods are reported.

### Data analysis

In order to quantify the healthfulness and type of checkout foods, we determined ‘checkout food exposures’. As in a previous study [14], this was defined as the total number of different product lines displayed across all possible ‘checkout journeys’. A checkout journey was defined as a route through the compulsory checkout area that shoppers had to pass through to pay for their goods [14]. In most stores numerous checkout journeys were possible due to multiple payment points and queuing areas shared between payment points. Any food lines displayed in a shared queuing area were multiple counted to reflect the total number of checkouts and hence possible different routes from the start of the queue to each payment point. For example, a chocolate product displayed in a shared queuing area leading to five payment points, was counted five times: once for each of the five possible checkout journeys.

The distributions of all variables in the dataset were not normally distributed, so non-parametric methods were used throughout. Simple descriptive statistics (e.g. number, median, and range) of foods per checkout journey and proportion of these that were less healthy were calculated overall, and by checkout food policy category. Non-parametric test for trends across ordered groups were used to explore differences in total number of foods per checkout journey, the proportion of these that were less healthy, and the proportion that were in each food group, by checkout food policy category.

In order to determine whether supermarkets adhered to their checkout food policies, we compared checkout food observed according to policy category. Depending on how vague policies were, this required some subjective interpretation of policies. In all cases we aimed to be pragmatic and consistent in our interpretations. KTE, in close discussion with JA, interpreted policies to develop algorithms for comparing observed checkout foods with policies. Supermarket-specific data was used to determine whether foods inconsistent with policies were present or not. We compared the proportion of checkout journeys with and without foods that were inconsistent with policies according to policy category using Fisher’s exact.

All analyses were conducted in STATA version 14.2 (StataCorp LP). The significance level was set at α = 0·05.

## Results

Within the nine supermarket groups included we found 14 store formats.

### Supermarket policies on checkout foods

The details of checkout food policies are described in Table 1. Across the 14 store formats, two had no checkout food policy, six of the policies were categorised as ‘clear and consistent’ and the remaining six as ‘vague or inconsistent’.

### Does the healthfulness and type of checkout food vary according to the presence or nature of checkout food policies?

In-store observations were conducted in a total of 69 stores across the 14 store formats – five stores of each of 13 formats and four of the remaining format as there were only four stores belonging to this store format located in the East of England region. A total of 9141 checkout food exposures were observed across 1044 checkout journeys. Table 2 shows the number of checkout journeys, percentage of checkout journeys without checkout foods, food exposures per checkout journey, and the percentage of these that were less healthy foods overall and across policy categories.

**Table 2:** Healthfulness of checkout foods by checkout food policy category

	All supermarkets	Checkout food policy status			
		Clear and consistent	Vague or inconsistent	No policy	*p* for trend
Total checkout journey, n	1044	430	496	118	–
Total checkout food exposures, n	9141	1919	5464	1758	–
% of checkout journey with no checkout food, median [range]	54 [0,100]	72 [0,100]	38 [0,100]	39 [0,100]	< 0.001
Food exposures per checkout journey, median [range]	17 [0;148]	13 [0;29]	15 [0;148]	39 [0;86]	< 0.001
% of foods exposures per checkout journey that were less healthy, median [range]	49 [0;100]	35 [0;50]	57 [0;100]	90 [0;100]	< 0.001

Overall there was a median of 17 foods per checkout journey, of which a median of 49% were less healthy. Comparable figures for supermarkets with a clear and consistent, vague or inconsistent, or no policy were 13 (35%), 15 (57%) and 39 (90%) respectively. We found trends towards increasing number of food exposures per checkout journey and increasing percentage of these that were less healthy when checkouts were ordered by policy category from clear and consistent through vague and inconsistent to no policy (ps < 0.001). In total, 564 out of 1044 checkout journeys (54%) had no food on display.

There was a trend towards a lower proportion of checkout journeys with no checkout foods when checkouts were ordered by policy category from clear and consistent through vague and inconsistent to no policy (*p* < 0.001). There was substantial variation between stores, formats and groups in food exposures per checkout journey and the percentage of these that were less healthy.

The number and percentage of checkout journeys that included foods in each food group are shown in Table 3 overall and by policy category. Overall, the most frequently represented food groups were sugar-free confectionery, sugary confectionery, and nuts & seeds.

**Table 3:** Type of checkout foods (grouped by NDNS food groups) by checkout food policy category

NDNS food group	Products included	Checkout journeys where food group present, n (%)				*p* for trend
		All supermarkets	Checkout food policy category			
			Clear and consistent	Vague or inconsistent	No policy	
Biscuits	Cream crackers, flapjacks, breadsticks, oatcakes, rice cakes, crispbread, cereal bars & protein snack bars, ice cream cornet/wafers, gluten free biscuits	291 (27.9)	63 (14.7)	87 (17.5)	27 (22.9)	< 0.001
Bottled water, carbonated or still		106 (10.2)	53 (12.3)	49 (9.9)	4 (3.4)	0.001
Buns, cakes, pastries & fruit pies	Purchased/retail buns, cakes or pastries; Danish pastries, currant bun, doughnuts, American muffins, Eccles cakes, Bakewell tarts, jam tarts, scones (sweet and savoury), sponge cakes, fruit cakes, eclairs, fruit loaf, malt loaf, gateaux, pastry, mince pies, sponge fingers, scotch pancakes, croissants, custard tart, lemon meringue pie, egg custard, caramel shortcake	45 (4.3)	0 (0)	23 (4.6)	19 (16.1)	< 0.001
Chocolate confectionery	Chocolate bars, filled bars, assortments, carob, diabetic and low calorie chocolate, traditional snack bars	241 (23.1)	0 (0)	165 (33.3)	44 (37.3)	< 0.001
Crisps & savoury snacks	All potato and cereal based snacks, popcorn (not sweet), twiglets, pretzels, pork scratchings, pea snacks, rice snacks, meat based snacks	219 (21.0)	50 (11.6)	55 (11.1)	29 (24.6)	< 0.001
Dried fruit	Dried fruit without added sugar, dried fruit with sugar, banana chips	160 (15.3)	72 (16.7)	68 (13.7)	20 (16.9)	0.59
Fresh fruit		2 (0.2)	2 (0.5)	0 (0)	0 (0)	0.13
High fibre breakfast cereals		15 (1.4)	0 (0)	0 (0)	15 (12.7)	< 0.001
Nuts & seeds	Fruit and nut mixes, coconut, salted peanuts, nut butters, tahini, bombay mix, snack bars based on dried fruit & nuts	369 (35.3)	88 (20.5)	92 (18.5)	23 (19.5)	0.34
Soft drinks, carbonated/still		47 (4.5)	3 (0.7)	23 (4.6)	6 (5.1)	< 0.001
Soft drinks, diet		67 (6.4)	14 (3.3)	49 (9.9)	4 (3.4)	0.06
Sugary confectionery	Boiled sweets, gums, pastilles, fudge, chews, mints, rock, liquorice, toffees, chewing gum, sweet popcorn, ice lollies (not ice cream), nougat, halva	326 (31.2)	36 (8.4)	213 (42.9)	77 (65.3)	< 0.001
Sugar-free confectionery	Sugar-free versions of products listed under ‘sugary confectionery’	377 (36.1)	123 (28.6)	237 (47.8)	17 (14.4)	0.57
White bread	All types of bread & bread products made with white wheat flour	11 (1.1)	0 (0)	3 (0.6)	8 (6.8)	< 0.001
Soup		1 (0.1)	0 (0)	1 (0.2)	0 (0)	0.651

There were trends in the proportion of checkout journeys that included foods in a number of food groups when checkouts were ordered by policy category from clear and consistent through vague and inconsistent to no policy (see Additional file 1: Table S1). Typically, these identified greater likelihood of foods in ‘less healthy’ food groups as strength of policy decreased, including: biscuits; buns, cakes, pastries & fruit pies; chocolate confectionery; crisps & savoury snacks; soft drinks, carbonated & still; and sugary confectionery. Only bottled water showed the opposite trend. There were no significant trends among dried or fresh fruits, nuts or seeds, or soups.

### Do supermarkets adhere to their checkout food policies?

Table 4 shows how we interpreted the policies described in Table 1 in order to determine supermarkets’ adherence to these policies. The percentage of relevant checkout journeys that were adherent with each policy are also shown in Table 4. Overall 155 (16.7%) checkout journeys were not adherent with the checkout food policies for the store in which they were located. All except one of the stores with clear and consistent policies were fully adherent to these. There was a significant difference in the proportion of checkout journeys that were adherent to relevant policies between supermarkets with clear and consistent policies and those with vague or inconsistent policies (*p* = 0.015). However, there were a number of individual shops within the groups with vague or inconsistent policies that fully adhered to these, and non-adherence may only have reflected one, amongst many, foods inconsistent with a policy.

**Table 4:** Supermarkets’ adherence to their own checkout food policies

Supermarket group	Store format	Policy category	Our interpretation of policy for determination of adherence	Checkout journeys with food inconsistent with policies, n (%)
1	Hypermarket	Clear and consistent	Only food that counts as 5-a-day, has no ‘red’ traffic light ratings, is in a pack of 50 g or less, or is deemed a ‘healthier’ snack by Department of Health	0
	Large supermarket	Clear and consistent	Only food that counts as 5-a-day, has no ‘red’ traffic light ratings, is in a pack of 50 g or less, or is deemed a ‘healthier’ snack by Department of Health	0
	Small supermarket	Clear and consistent	Only food that counts as 5-a-day, has no ‘red’ traffic light ratings, is in a pack of 50 g or less, or is deemed a ‘healthier’ snack by Department of Health	3 (5.1)
	Convenience store	Clear and consistent	Only food that counts as 5-a-day, has no ‘red’ traffic light ratings, is in a pack of 50 g or less, or is deemed a ‘healthier’ snack by Department of Health	0
2	Large supermarket	Vague or inconsistent	No foods in the sugary confectionery or chocolate confectionery food groups in main checkouts (staffed belt checkouts)	2 (2.4)
3	Large supermarket	Vague or inconsistent	No foods in the sugary confectionery, chocolate confectionery or soft drinks, carbonated/still food groups in 1/3 of checkouts	Metric not applicable given focus on 1/3 checkouts^1^
4	Large supermarket	Vague or inconsistent	No foods in the sugary confectionery or chocolate confectionery food groups in main checkouts (directly at the till)	Metric not applicable given difference in definition of checkout area^2^
5	Large supermarket	Clear and consistent	Only foods in the nuts & seeds, dried fruit, sugar-free confectionery, soft drinks, diet and bottled water food categories; as well as crispbread/flatbread, popcorn and rice snacks, savoury vegetable snacks, snack bars based on dried fruit & nuts, cereals or protein	0
7	Large supermarket	Vague or inconsistent	No foods in the sugary confectionery or chocolate confectionery food groups	14 (17.9)
	Convenience store	Vague or inconsistent	No foods in the sugary confectionery or chocolate confectionery food groups	3 (11.5)
8	Large supermarket	Clear and consistent	Only foods in the nuts & seeds, dried fruit, sugar-free confectionery, soft drinks, diet and bottled water food categories; as well as crispbread/flatbread, popcorn and rice snacks, savoury vegetable snacks, snack bars based on dried fruit & nuts, cereals or protein	0
9	Convenience store	Vague or inconsistent	No foods in the sugary confectionery, chocolate confectionery, crisps & savoury snacks, biscuits or buns, cakes, pastries & fruit pies food groups (franchise stores excluded)	6 (60.0)

^1^Two out of the five included stores had no checkout foods; the other three had 78.4, 84.0 and 100% of checkout journeys with food inconsistent with policies hence these three stores were non adherent with the policy to have 1/3 of checkouts ‘guilt free’

^2^Using our definition of checkout area, 95 (79.2%) of checkouts contained food inconsistent with policy

## Discussion

### Summary of principal findings

This is the first study we are aware of to document how checkout food varies according to the nature of supermarket checkout food policies, and whether these policies are adhered to.

Across nine supermarket groups representing 90% of the UK grocery market, we found 14 store formats. Of these formats, six had a clear and consistent checkout food policy, six had a vague or inconsistent policy, and two had no policy.

We found a median of 17 foods per checkout journey, of which a median of 49% were less healthy. Checkout journeys in stores with clear and consistent checkout policies had fewer foods and a lower proportion of these were less healthy than in other stores.

Checkout journeys most often figured checkout food in the following food groups: sugar-free confectionery, sugary confectionery, and nuts & seeds. Food groups typically considered ‘less healthy’ were more likely to be available at checkouts in stores without a checkout food policy.

All except one of the stores with clear and consistent policies appeared to fully adherent to these. This did not appear to be the case in stores with vague or inconsistent policies.

### Strengths and weaknesses

A priori, we thought it likely that checkout food and adherence to checkout food policies may vary by store format. As such, we used stratified random sampling to include similar numbers of stores across different store formats. This means that our sample is not representative of the UK grocery market as a whole. However, given the homogeneity of the UK grocery market and our inclusion of supermarket groups representing 90% of market share, our findings are likely to be generalisable across the UK. Whilst our descriptive findings may not be more widely generalisable, our finding that clear and consistent checkout food policies are associated with more healthful checkout foods and are more likely to be adhered to may well be.

Our classification of supermarket policies was post hoc and combines both clarity and consistency. Re-classification according to either just clarity or just consistency led to similar trends and unchanged conclusions (data now shown).

Our data collection was conducted over 4 months including Easter. Checkout food may show seasonal variations. It is also possible that there are seasonal variations in the impact of policies on checkout food and supermarket adherence to their policies.

We used the same method for data collection as used in a previous study [14], which has been shown to have high inter-rater reliability. The NPM groups foods into just two categories. Clearly foods and diets show more variation in their healthfulness than this. We accounted for this with the use of food groups. Both the NPM and the food groups we used were derived from current UK policy contexts. However, both tools necessarily collapse a wide range of food into a small number of groups and so mask wide variation in foods observed. More detailed variations by product, rather than food, group can be found in Additional file 1: Table S1.

In the majority of cases, we obtained nutritional information on checkout foods from manufacturers’ and supermarkets’ website, or packaging. This data may be subject to some error.

We made substantial efforts to clarify supermarkets’ checkout food policies – often phoning, emailing and writing multiple times to multiple different contacts. However, we were not able to obtain the information we sought in all cases directly from supermarkets and were forced to rely on secondary sources. Further, we looked for national policies but it is possible that there may be more localised policies in place. Thus, there may be some error in our assessment of policy content. Further, when assessing adherence to policies we were forced to interpret some vague statements. We cannot be certain we have interpreted all policies as they were intended and our results on adherence should be interpreted with caution.

Our study involved observing the range of checkout food available, not whether this was purchased or consumed. We cannot, therefore, make any conclusions about the impact of checkout food, or checkout food policies, on customers’ purchasing, consumption, or total diet. As our study was cross-sectional and observational, we are also not able to conclude with certainty that differences in checkout food seen between supermarkets with different policies are attributable to those policies.

### Comparison of findings to previous studies

Previous studies have reported that very large proportions of UK checkout food are less healthy. In 2014, 78% of checkout food in UK convenience supermarkets was considered less healthy [15]. Likewise in 2015, more than 80% of checkout foods in 32 UK non-food stores was less healthy [14]. Our results for supermarkets without checkout food policies were similar (median 90% of checkout food exposures were less healthy). However, our overall total of 49% of checkout food exposures being less healthy is less than previously reported in the UK. This may reflect known variations in checkout food across time, place or both [8, 10, 21]. Indeed, during our search for information on supermarket checkout food policies we found evidence that a number of supermarkets have introduced, dropped or altered policies over the last 5 years [20, 40, 41].

We are not aware of any previous research exploring the impact of supermarket checkout food policies with which to compare our other findings.

### Interpretation of findings

We found that supermarket customers are likely to be exposed to substantial volume of checkout food. Thus, the checkout clearly remains an area where retailers believe there is opportunity to prompt purchases. However, we also found a high number of customer journeys with no checkout food, indicating that not all supermarkets feel the necessity to make use of this opportunity. Overall, where food was available nearly half of checkout food exposures were healthier, indicating that some retailers do find it feasible to offer healthier checkout food at least in some cases.

We found that the type and healthfulness of checkout foods varied significantly according to the presence and nature of checkout food policies. Healthfulness was greater in supermarkets with vague and inconsistent policies than in those with no policy, but greatest in those with clear and consistent policies. This suggests that having any policy concerning checkout food is likely to be associated with improvements in the healthfulness of checkout food. Our findings indicate that encouraging those supermarkets that have not yet developed a policy, or who have developed only a vague or inconsistent policy, to develop a clear and consistent policy may further improve checkout food healthfulness.

We also found an indication that supermarkets with clear and consistent checkout food policies were more likely to fully adhere to this. This might explain why clear and consistent policies were associated with greater decrease in the proportion of less healthy checkout food than vague or inconsistent policies. This is another reason to encourage supermarkets to develop such clear and consistent policies on checkout food.

The content of checkout food policies varied substantially between supermarket groups and between different stores formats within the same group. We also observed large variations in checkout food types and adherence to policies within the same store formats (data not shown). This suggests that there may be variation in how local store managers are made aware of, or asked to implement, strategic decisions from headquarters concerning checkout food. Clear and consistent policies appear to limit such variation in implementation and we noticed some indication that these supermarkets were more likely to display products that appeared to have been specifically designed for checkout areas (e.g. small packages of dried fruits and/or nuts).

Intervention studies that manipulate checkout food conclude that to change customers’ purchases substantially, healthy alternative checkout food must match consumers’ shopping habits and expectations for suitable snacks [26–28]. Fresh fruit is commonly purchased and likely to be placed in the shopping basket before reaching the checkout. In contrast, snack-like products are less commonly purchased, particularly if they are not located in prominent store locations. This means that healthier snack-like products may have greater potential as healthier checkout food than fresh fruit [28]. This seems to be reflected in UK experience, where several early announcements on healthier checkout foods mentioned fresh fruit as replacements [20, 40–42]. However, we found fresh fruit was only represented in 0.2% of checkout journeys suggesting that initial attempts to place fresh fruit at checkouts may have failed.

Healthier checkout food options should also reflect local food culture [28]. Finding the best alternatives that maximise supermarket sales and profits is likely to require product development and testing. We found a large number of different checkout food options that were nutritionally similar (e.g. many different dried fruit and nut mixes – data not shown). By making a range of different products available, supermarkets encourage consumers to try new foods [43]. This may also indicate ongoing product development and testing.

### Implications and unanswered questions

Public health policymakers and advocacy groups may want to continue to encourage those supermarkets without a clear and consistent policy to develop one and make it publically available both in the UK and internationally. Some civil society groups have called for government regulation on checkout food [11, 20]. Although our research did not include non-food stores, such as book, toy, and fashion stores, there is a rationale for also encouraging non-food stores to develop such policies.

Further research is required to understand what the impact of supermarket checkout food policies is on purchasing, consumption and total diet. There are also unanswered questions concerning what the optimal healthier checkout foods are to maximise both customers’ diets and profits [30].

Online grocery shopping now accounts for around 7% of market share in the UK [44]. Although different from in-store environments, there are still opportunities for encouraging impulse purchasing on-line. There is currently little information on on-line food impulse marketing and purchasing. We are not aware of any UK supermarkets that have developed a healthier policy in this area and this may be an increasingly important area for future research and policymaking.

## Conclusion

Most UK supermarkets have a checkout food policy, but only around half of these are clear and consistent. Customers shopping in supermarkets with clear and consistent policies are likely to be exposed to fewer checkout foods, and a lower proportion of these foods are likely to be less healthy than in supermarkets with a vague or no policy. Most supermarkets with a clear and consistent policy appear to adhere well to it. Supermarkets with a vague or inconsistent policy appear to adhere less well.

Further research is required to understand what the impact of these policies is on purchasing and consumption of checkout food, as well as total diet. Public health policymakers and advocacy groups may want to encourage those supermarkets and non-food stores without a clear and consistent policy to develop one.

## Additional file

Additional file 1: **Table S1.** Type of checkout foods (grouped by product groups) by checkout food policy category (DOCX 16 kb)

## Abbreviations

NDNS: National Diet & Nutrition Survey
NPM: Nutrient Profile Model
