# Supplementary material for: The nature of UK supermarkets’ policies on checkout food and associations with healthfulness and type of food displayed: cross-sectional study
Source: Int J Behav Nutr Phys Act. 2018 Jun 11;15:52. doi: 10.1186/s12966-018-0684-2 (PMC5996483; doi:10.1186/s12966-018-0684-2)
Supplement: Supplementary file 1 — Table S1. Type of checkout foods (grouped by product groups) by checkout food policy category (DOCX 16 kb) [file 12966_2018_684_MOESM1_ESM.docx]

Table S1: Type of checkout foods (grouped by product groups) by checkout food policy category

|  | **Commitments on healthy checkout foods** | | |  |
| --- | --- | --- | --- | --- |
| **Product groups** | **Absent** | **Vague or inconsistent** | **Clear and consistent** | **P for trend** |
|  | **Checkout journeys with food present**  **N (%)** | **Checkout journeys with food present**  **N (%)** | **Checkout journeys with food present**  **N (%)** |  |
| Biscuits | 23 (19.5) | 11 (2.2) | - | <0.001 |
| Bottled water, carbonated/still | 4 (3.4) | 49 (9.9) | 53 (12.3) | 0.007 |
| Chewing gum, sugar-free | 17 (14.4) | 203 (40.9) | 103 (24.0) | 0.29 |
| Chocolate | 43 (36.4) | 146 (29.4) | - | <0.001 |
| Confectionery | 33 (28.0) | 154 (31.0 ) | 3 (0.7) | <0.001 |
| Crackers | 19 (16.1) | 4 (0.8) | - | <0.001 |
| Crispbread/flatbread | - | 3 (0.6) | 58 (13.5) | <0.001 |
| Crisps | 17 (14.4) | 41 (8.3) | 12 (2.8) | <0.001 |
| Dried fruit | 20 (16.9) | 68 (13.7) | 72 (16.7) | 0.59 |
| Fresh fruit | - | - | 2 (0.5) | 0.133 |
| High fibre breakfast cereals | 15 (12.7) | - | - | <0.001 |
| Meat-based snacks | - | 3 (0.6) | - | 0.43 |
| Mix nut/fruit candy | 19 (16.1) | 39 (7.9) | 5 (1.2) | <0.001 |
| Mix of nuts, seeds, dried fruit | 16 (13.6) | 71 (14.3) | 80 (18.6) | 0.07 |
| Nuts | 20 (16.9) | 47 (9.5) | 46 (10.7) | 0.24 |
| Pastilles, sugar-free | - | 34 (6.9) | 20 (4.7) | 0.41 |
| Savoury popcorn & rice snacks | 23 (19.5) | 14 (2.8) | 38 (8.8) | 0.18 |
| Snack bars, cereal | 16 (13.6) | 72 (14.5) | 16 (3.7) | <0.001 |
| Snack bars, fruit and nut based | 20 (16.9) | 40 (8.1) | 29 (6.7) | 0.003 |
| Snack bars, protein | 15 (12.7) | 34 (6.9) | 20 (4.7) | 0.003 |
| Snack bars, traditional | 23 (19.5) | 29 (5.8) | - | <0.001 |
| Soft drinks, diet | 4 (3.4) | 49 (9.9) | 14 (3.3) | 0.06 |
| Soup | - | 1 (0.2) | - | 0.65 |
| SSB | 6 (5.1) | 23 (4.6) | - | <0.001 |
| SSB, reduced sugar | - | 15 (3.0) | 3 (0.7) | 0.39 |
| Sweet baked goods | 19 (16.1) | 15 (3.0) | - | <0.001 |
| Sweet bread types | 1 (0.8) | 10 (2.0) | - | 0.05 |
| Sweet popcorn & rice snacks | 25 (21.2) | 20 (4.0) | 28 (6.5) | 0.001 |
| Vegetable savoury snacks | 16 (13.6) | 29 (5.8) | 26 (6.0) | 0.037 |
| White bread | 8 (6.8) | 3 (0.6) | - | <0.001 |

SSB: Sugar sweetened beverages.
